# Neonatal outcomes after preterm birth by mothers’ health insurance status at birth: a retrospective cohort study

**DOI:** 10.1186/1472-6963-13-40

**Published:** 2013-02-04

**Authors:** Kristjana Einarsdóttir, Fatima A Haggar, Amanda T Langridge, Anthony S Gunnell, Helen Leonard, Fiona J Stanley

**Affiliations:** 1 Telethon Institute for Child Health Research, Centre for Child Health Research, University of Western Australia, 100 Roberts Road, Subiaco, WA, 6008, Australia; 2 Centre for Health Services Research, School of Population Health, The University of Western Australia, 35 Stirling Highway Crawley, Perth, WA, 6009, Australia; 3 Health and Wellness Institute, Edith Cowan University, 270 Joondalup Drive, Joondalup, WA, 6027, Australia

**Keywords:** Health insurance, Preterm birth, Neonatal outcomes, Apgar score, Neonatal resuscitation

## Abstract

**Background:**

Publicly insured women usually have a different demographic background to privately insured women, which is related to poor neonatal outcomes after birth. Given the difference in nature and risk of preterm versus term births, it would be important to compare adverse neonatal outcomes after preterm birth between these groups of women after eliminating the demographic differences between the groups.

**Methods:**

The study population included 3085 publicly insured and 3380 privately insured, singleton, preterm deliveries (32–36 weeks gestation) from Western Australia during 1998–2008. From the study population, 1016 publicly insured women were matched with 1016 privately insured women according to the propensity score of maternal demographic characteristics and pre-existing medical conditions. Neonatal outcomes were compared in the propensity score matched cohorts using conditional log-binomial regression, adjusted for antenatal risk factors. Outcomes included Apgar scores less than 7 at five minutes after birth, time until establishment of unassisted breathing (>1 minute), neonatal resuscitation (endotracheal intubation or external cardiac massage) and admission to a neonatal special care unit.

**Results:**

Compared with infants of privately insured women, infants of publicly insured women were more likely to receive a low Apgar score (ARR = 2.63, 95% CI = 1.06-6.52) and take longer to establish unassisted breathing (ARR = 1.61, 95% CI = 1.25-2.07), yet, they were less likely to be admitted to a special care unit (ARR = 0.84, 95% CI = 0.80-0.87). No significant differences were evident in neonatal resuscitation between the groups (ARR = 1.20, 95% CI = 0.54-2.67).

**Conclusions:**

The underlying reasons for the lower rate of special care admissions in infants of publicly insured women compared with privately insured women despite the higher rate of low Apgar scores is yet to be determined. Future research is warranted in order to clarify the meaning of our findings for future obstetric care and whether more equitable use of paediatric services should be recommended.

## Background

Infants born preterm (<37 weeks gestation) have a high risk of mortality and morbidity with the risk being the highest in those born before 32 weeks [1,2]. For example, a survival rate of almost 100% can now be expected in infants born after 32 weeks gestation [1]. Despite the high survival rate in those born between 32 and 36 weeks gestation compared with those born earlier, they remain a high risk group compared with term births [3]. Furthermore, these births are much more common than births of earlier gestation and as such the public health impact of this group is high [3].

Women in Australia can choose to give birth with public or private health insurance, depending on their financial means. Publicly insured women receive free services with the care provided by rostered midwives, residents, registrars and staff obstetricians in public hospitals. Privately insured women however receive antenatal and hospital care at a subsidized cost, with the antenatal care led by private obstetricians and women delivering in either public or private hospitals. Evidence suggests that publicly insured women are generally less healthy and in more need of health care services than other women [4-12]. This appears to be related to poor outcomes for infants born at term in Australia [13]. Eliminating the demographic differences between publicly insured and privately insured women would therefore seem important in order to investigate whether there are true differences in neonatal outcomes between these groups of women. Furthermore, the difference in nature and risk of preterm versus term births, illustrates the importance of focusing on preterm births as a group in its own right.

In this study, we aimed to compare neonatal outcomes after preterm birth between publicly and privately insured women in Western Australia. We used propensity score methods to circumvent the problems with traditional methods of matching and covariate adjustment when the number of confounders is high between the comparison groups [11,12], as is the case in this study. This analytical method has recently begun to be used within obstetrics to reduce or eliminate fundamental differences between comparison groups [14,15]. First, we estimated the propensity of being a privately insured woman based on the demographics and pre-existing medical conditions. Then the estimated propensity scores were used to individually match publicly insured women with privately insured women. Lastly, we compared the differences in adverse neonatal outcomes between the matched groups using conditional log-binomial regression.

## Methods

### Ethics information

The use of de-identified, administrative health data for this study without patient consent was approved by the Human Research Ethics Committee of the Western Australian Department of Health. This study was performed in accordance with the Declaration of Helsinki.

### Data information

In this study we used linked, administrative data provided by the Data Linkage Branch at the Western Australian Department of Health. We selected the study cohort (n = 6,465) from the Midwives Notification System, which was linked with the Hospital Morbidity Data Collection and the Registry of Congenital Anomalies. The midwives data records information on all live or stillborn infants at least at 20 weeks gestation or with birth weight of at least 400 gr. The hospital data included hospital admission information for each birth that occurred in public or private hospitals during 1998–2008 and the Registry of Congenital Anomalies included information on whether an infant had a minor or a major birth defect. We restricted our study population to singleton, preterm births (32–36 weeks gestation), where the infant was live-born and without birth defects and the mother delivered in a non-tertiary hospital.

Included in the midwives data was information on SE disadvantage, obtained from the Index of Relative Socio-Economic Disadvantage (IRSD), which is calculated from Australian Census information according to collection districts. The midwives data also contained information on the Accessibility/Remoteness Index of Australia (ARIA+). The IRSD and ARIA values were based on the 2001 and 2006 Censuses and were assigned to each birth admission based on maternal area of residence at the time of birth. The hospital data provided information on funding source and hospital type at the time of delivery, with privately insured women being defined as those funded by private health insurance or who were self-funded and publicly insured women defined as those funded under the Australian Health Care Agreements or the Reciprocal Health Care Agreements.

Privately insured people can be treated in either public or private hospitals in Australia, whereas publicly insured Australians could only be treated in public hospitals until 1996, when public patient facilities were established at a large private hospital in the Perth metropolitan area [11]. We only included publicly insured women who delivered in public hospitals and privately insured women who delivered in private hospitals in this study in order to ensure homogeneity of each comparison group.

### Statistical analysis

The propensity scores were generated by a logistic regression model referred to as the propensity model. The scores represented the probability of delivering as a privately insured woman in a private hospital given the maternal demographics - including squared terms and interactions – that were included as covariates [16]. The following maternal demographics (as well as 11 interaction terms and one square term for age) were significant in the forward selection model (p < 0.05) and were included in the propensity model: Maternal age (continuous), parity (ordinal; 14 levels), marital status (categorical; married/other), ethnicity (categorical; eight levels), smoking during pregnancy (categorical; yes/no), SE disadvantage (categorical; five levels), residential remoteness (categorical; five levels), pre-existing diabetes mellitus (categorical; yes/no), previous caesarean (categorical; yes/no) and year of birth (ordinal; 11 levels).

In order to match each privately insured woman with each publicly insured woman within 0.25 standard deviation of the logit of the propensity score of the identified privately insured woman, we conducted 1:1 matching without replacement [17]. The balance of the covariates between the comparison groups before and after matching was assessed using chi square tests of independence with those significantly different (p < 0.05) indicating imbalance of covariates. We used conditional log-binomial regression model of the matched sets to estimate relative risk and 95% confidence intervals for the risk of adverse infant outcomes for publicly insured women delivering in public hospitals compared with privately insured women delivering in private hospitals. The infant outcomes assessed in this study included Apgar score less than 7 at five minutes after birth, time until establishment of unassisted breathing (>1 minute), neonatal resuscitation (endotracheal intubation or external cardiac massage) and neonatal admission to a special care nursery. The models were adjusted for antenatal risk factors including threatened abortion, threatened preterm labour, pre-eclampsia, placenta praevia and pre-labour rupture of membranes.

All analyses were performed using the statistical software SAS version 9.3 (SAS Institute Inc., Cary, NC, USA).

## Results

Our study population included 6,464 preterm births (32–36 weeks) where 47.7% of the mothers were publicly insured in a public hospital and 52.3% were privately insured in a private hospital at the time of birth. Table 1 presents the balance of maternal demographics between the publicly insured women (n = 3,085) and privately insured women (n = 3,380) before matching. All covariates were significantly different between the two groups, thus illustrating the high imbalance in demographic characteristics between the publicly and privately insured women.

**Table 1:** **Maternal demographics used for determination of the propensity score for 6,465**^**a **^**unmatched public and private deliveries**

	**Publicly insured women**	**Privately insured women**	
	**(n = 3,085)**	**(n = 3,380)**	**p-value**^**b**^
	Mean (±SD)	Mean (±SD)	
Maternal age (years)	27.0 (6.0)	32.2 (4.6)	<0.0001
	n (%)	n (%)	
Parity (2^nd^ + child)	1,739 (56.4)	1,563 (46.2)	<0.0001
Single/divorced/widowed	479 (15.5)	99 (2.9)	<0.0001
Indigenous	629 (20.4)	6 (0.2)	<0.0001
Smoking during pregnancy	1,286 (41.7)	209 (6.2)	<0.0001
SE disadvantage	1,832 (59.4)	517 (15.3)	<0.0001
Regional/remote residence	1,606 (52.1)	281 (8.3)	<0.0001
Diabetes mellitus	24 (0.8)	55 (1.6)	0.019
Previous caesarean	413 (13.4)	721 (21.3)	<0.0001

^a^ Restricted to singleton, pre-term births (32–36 weeks), in non-tertiary hospitals, resulting in live-born infants without birth defects.

^b^ ttest for means and Chi square test of independence for proportions.

Table 2 shows the balance of covariates after the 1:1 matching of publicly insured women with privately insured women was performed according to the propensity score of maternal demographics. We were able to individually match 1016 privately insured women with 1016 publicly insured women within 0.25 standard deviations of the logit of the propensity score. The table shows that all covariates were balanced between the two groups after matching as no statistically significant differences in proportions were evident. We were thus confident in that the two matched groups of women were highly similar with regard to their demographic characteristics.

**Table 2:** **Maternal demographics of the 2,032**^**a **^**public and private deliveries that were individually matched on the propensity score**

	**Publicly insured women**	**Privately insured women**	
	**(n = 1,016)**	**(n = 1,016)**	**p-value**^**b**^
	Mean (±SD)	Mean (±SD)	
Maternal age (years)	30.2 (5.1)	30.3 (5.0)	0.50
	n (%)	n (%)	
Parity (2^nd^ + child)	457 (45.0)	460 (45.3)	0.89
Single/divorced/widowed	59 (5.8)	52 (5.1)	0.49
Indigenous	4 (0.4)	5 (0.5)	0.74
Smoking during pregnancy	153 (15.1)	162 (15.9)	0.58
SE disadvantage	328 (32.3)	323 (31.8)	0.81
Regional/remote residence	222 (21.9)	224 (22.1)	0.91
Diabetes mellitus	6 (0.6)	3 (0.3)	0.32
Previous caesarean	147 (14.5)	146 (14.4)	0.95

^a^ Restricted to singleton, pre-term births (32–36 weeks), in non-tertiary hospitals, resulting in live-born infants without birth defects.

^b^ ttest for means and Chi square test of independence for proportions.

In Table 3 we show the differences in antenatal risk factors, gestation, mode of delivery and infant weight between the two matched groups. Threatened abortion (p = 0.006), pre-eclampsia (p < 0.0001) and placenta praevia (p = 0.0006) were more common in privately insured women, whereas threatened preterm labour (p = 0.008) and pre-labour rupture of membranes (p < 0.0001) were more common in publicly insured women. Also, privately insured women were more likely to delivery infants at lower gestation (p < 0.0001) and lower birth weight (p = 0.0002) and were twice as likely as publicly insured women to undergo pre-labour caesarean section (p < 0.0001).

**Table 3:** **Antenatal and delivery characteristics of the 2,032**^**a **^**public and private deliveries that were individually matched on the propensity score of maternal demographics**

	**Publicly insured women**	**Privately insured women**	**Degrees of**	
	**(n = 1,016)**	**(n = 1,016)**	**freedom**	**p-value**^**b**^
Antenatal risk factors	n (%)	n (%)		
Threatened abortion (<20 weeks)	63 (6.2)	96 (9.5)	1	0.006
Threatened preterm labour (<37w)	157 (15.5)	116 (11.4)	1	0.008
Urinary tract infection	41 (4.0)	28 (2.8)	1	0.11
Pre-eclampsia	76 (7.5)	153 (15.1)	1	<0.0001
Placenta praevia	15 (1.5)	40 (3.9)	1	0.0006
Placental abruption	24 (2.4)	23 (2.3)	1	0.88
Pre-labour rupture of membranes	259 (25.5)	183 (18.0)	1	<0.0001
Gestational diabetes	63 (6.2)	48 (4.7)	1	0.14
Gestation (weeks)				
32	9 (0.9)	21 (2.1)		
33	13 (1.3)	30 (3.0)		
34	43 (4.2)	145 (14.3)		
35	206 (20.3)	231 (22.7)		
36	745 (73.3)	589 (58.0)	4	<0.0001
Mode of delivery				
Unassisted vaginal	551 (54.2)	358 (35.2)		
Assisted vaginal	114 (11.2)	142 (14.0)		
Caesarean section in labour	196 (19.3)	192 (18.9)		
Pre-labour caesarean section	155 (15.3)	324 (31.9)	3	<0.0001
	Mean (±SD)	Mean (±SD)		
Infant weight (g)	2,764.6 (471.1)	2,687.4 (471.1)		0.0002

^a^ Restricted to singleton, pre-term births (32–36 weeks), in non-tertiary hospitals, resulting in live-born infants without birth defects.

^b^ ttest for means and Chi square test of independence for proportions.

^c^ Only deliveries with labour.

We compare the risk of adverse neonatal outcomes between the matched publicly and privately insured women in Table 4. Compared with delivering under private insurance in a private hospital, delivering under public insurance in a public hospital was associated with an increased risk of low Apgar scores in the infant five minutes after birth (RR = 2.50, 95% CI 0.97-6.44) and the infant taking longer than one minute to establish unassisted breathing (RR = 1.57, 95% CI = 1.22-2.02), while it was also associated with a reduced risk of admission to neonatal special care unit (RR = 0.83, 95% CI 0.80-0.87). The risk of low Apgar scores became statistically significant after adjustment for antenatal risk factors, but none of the other estimates were significantly altered after the adjustment (Table 4). Additionally adjusting for either mode of delivery, gestation or infant weight did not significantly alter any of the estimates (data not shown).

**Table 4:** **Risk of adverse neonatal outcomes after birth for 1,016**^**a **^**publicly insured women compared with 1,016**^**a **^**privately insured women that were individually matched on the propensity score of maternal demographics**

	**n**	**Unadjusted**	**Adjusted for antenatal factors**
	**public/private**	**RR (95% CI)**	**ARR (95% CI)**^**b**^
Low Apgar score at 5 mins <7^c^	15/6	2.50 (0.97–6.44)	2.63 (1.06–6.52)
>1 min to unassisted breathing^e^	137/87	1.57 (1.22–2.02)	1.61 (1.25–2.07)
Resuscitation^f^	12/10	1.20 (0.54–2.67)	---^d^
Admission to special care	96/251	0.83 (0.80–0.87)	0.84 (0.80–0.87)

^a^ Restricted to singleton, preterm births (32–36 weeks gestation), where the infant was live-born and without birth defects.

^b^ Adjusted for threatened abortion, threatened pre-term labour, pre-eclampsia, placenta-praevia, and pre-labour rupture of membranes.

^c^ Apgar score at 5 minutes = 0–6.

^d^ Too small sample size for reliable estimation.

^e^ >1 minutes until establishment of unassisted breathing.

^f^ Endotracheal intubation or external cardiac massage.

## Discussion

This study reports on the risk of adverse neonatal outcomes following preterm birth in publicly insured mothers that were individually matched with privately insured mothers based on their demographic characteristics. The results indicated that infants of publicly insured women of the same demographic characteristics as privately insured women were associated with an increased risk of low Apgar score and taking longer than one minute to establish unassisted breathing. Yet, they were less likely than infants of privately insured women to be admitted to a special care nursery for observation or treatment.

Using whole-population, routinely-collected, administrative medical/health data was a strength of this study as it minimised recall bias and loss-to-follow-up. We felt confident in using the midwives data for study population ascertainment as it is a statutory requirement in Western Australia that it records information on all births occurring on or after 20 weeks gestation or for infants born with birth weight of at least 400 g. Furthermore, it is a legislative requirement that the Department of Health records information on all hospital admissions and separations from all hospitals in the State. Information on funding source at the time of birth for the mother was available for 99% of all hospital births included in this study. Despite the number of strengths, a few limitations warrant attention. For example, using this type of data did create some limitations with respect to information availability as we were unable to adjust for body mass index prior at beginning of pregnancy or gestational weight gain. These factors and others can cause confounding as they can be associated with both patient status and maternal or infant outcomes. Despite that we were able to include in the propensity model a number of other factors known to be associated with body mass index during pregnancy, including socio-economic disadvantage [18], we may not have been able to completely eliminate residual confounding in our study.

Evidence suggests that people with private health insurance are healthier and in less need of health care services than those who do not have private health insurance [8]. Yet, private health insurance holders have been consistently shown to consume a disproportionately large share of health care services [8]. For example, women with private health insurance have less likelihood of smoking whilst pregnant [10], better smoking hygiene around their baby [12], less disturbances of mood during pregnancy and following childbirth [11], less risk of newborn encephalopathy [19] and less likelihood of hypertension, threatened preterm labour, antepartum haemorrhage, threatened labour and excessive vomiting during pregnancy [9]. Nevertheless, women with private health insurance have more likelihood of episiotomy [20], a higher probability of caesarean or instrumentally assisted delivery [21], a higher risk of forceps or vacuum delivery and of other obstetric interventions such as epidural anaesthesia, induction or augmentation [7]. These differences in demographic and health characteristics are likely to relate to poor neonatal outcomes. For example, a previous studies for term births found an increased risk of all adverse birth outcomes for publicly insured women compared to privately insured women [13].

Despite having minimised any differences in maternal demographic characteristics and pre-existing medical conditions between publicly insured and privately insured women, our results indicated an increased risk of low Apgar scores and taking more than one minute to establish unassisted breathing for infants of publicly insured women. Apgar scores at 5 minutes have reported to be better in healthy infants born by elective caesarean than in other infants [22] and our results indicated that privately insured women were almost twice as likely as publicly insured women to deliver by pre-labour caesarean section following a preterm birth. However, adjusting for mode of delivery did not change our results and it is therefore more likely that the higher risk of low Apgar score in publicly insured women in public hospitals is either due to residual confounding in our data or practise differences between public and private hospitals in Western Australia. For example, in the case of serious delivery complications, the situation in the delivery room may become tense and the assignment of Apgar scores may be incomplete or delayed [23]. As the Apgar score reflects the heart rate, breathing, appearance and responsiveness of the newborn infant at one minute and five minutes after delivery [24], delayed assignment may result in better scores being assigned since the infant has had time to recover from the birth trauma. As we did not have information on the actual time of the Apgar score assignment, we are unable to decipher whether a difference in Apgar score assignment between public and private hospitals may explain our results.

In contrast to our findings of increased risk of respiratory morbidity in preterm infants of publicly insured women, our results also indicated that infants of publicly insured women were less likely to be admitted to neonatal special care nursery than infants of privately insured women. This finding could not be explained by differences in maternal characteristics, antenatal risk factors, gestation or mode of delivery and thus could be a consequence of actual practice differences between public and private hospitals in Western Australia. Evidence suggests that discriminating between infants needing special care and those who do not is problematic [25] and that clinical guidelines vary greatly between special care nurseries in the initial management of infants with respiratory distress and in the thresholds to transfer to a neonatal intensive care unit [26]. In Australia, an admission to a special care unit can create additional funds for the hospital if the mother is a privately insured at the time of birth [27] since infants become separate fee paying patients from the mother once they are admitted to a special care unit in Australian hospitals. This is different for women who are admitted under public insurance, as the costs for admission of their infants is borne by the hospital. As a result, there may be an incentive for private hospitals to encourage the admission of borderline infants for observation in a special care unit. This and the fact that we found an increased risk of respiratory morbidity despite a lower likelihood of special care admission for infants of publicly insured women raises the possibility that some infants of privately insured women are offered admission unnecessarily [28] and/or that some infants of publicly insured women are not receiving adequate postnatal care.

## Conclusions

In conclusion, our findings indicate that after taking into account maternal characteristic and antenatal risk differences between publicly insured and privately insured women giving birth prematurely in Western Australia, infants of publicly insured women are more likely than infants of privately insured women to be assigned low Apgar scores, but are nevertheless less likely to be admitted to a special care nursery. Further research is warranted in order to clarify the meaning of our findings for future obstetric care and whether more equitable use of paediatric services should be recommended.

## Abbreviations

RR: Risk Ratio; ARR: Adjusted Risk Ratio.

## Competing interests

The authors declare no competing interests.

## Authors’ contributions

KE initiated the research, designed the study, analysed and interpreted the data, and wrote the article. FAH and AL gave advice on the statistical methods used for analysis and discussed ideas. ASG gave advice and commented on the manuscript. HL and FJS supervised the work, gave advice and contributed to the paper. All authors revised the paper critically for important intellectual content and approved it to be published.

## Pre-publication history

The pre-publication history for this paper can be accessed here:

http://www.biomedcentral.com/1472-6963/13/40/prepub
